# Factors related to health civic engagement: results from the 2018 National Survey of Health Attitudes to understand progress towards a Culture of Health

**DOI:** 10.1186/s12889-020-08507-w

**Published:** 2020-05-07

**Authors:** Tamara Dubowitz, Christopher Nelson, Sarah Weilant, Jennifer Sloan, Andy Bogart, Carolyn Miller, Anita Chandra

**Affiliations:** 1 RAND Corporation, 4570 Fifth Avenue, Suite 600, Pittsburgh, PA 15213 USA; 2 Robert Wood Johnson Foundation, Princeton, NJ USA; 3 RAND Corporation, Social & Economic Wellbeing, Santa Monica, USA

**Keywords:** Civic engagement, Health civic engagement, Sense of community, Community health investment

## Abstract

**Background:**

Civic engagement, including voting, volunteering, and participating in civic organizations, is associated with better psychological, physical and behavioral health and well-being. In addition, civic engagement is increasingly viewed (e.g., in Robert Wood Johnson Foundation’s Culture of Health action framework) as a potentially important driver for raising awareness of and addressing unhealthy conditions in communities. As such, it is important to understand the factors that may promote civic engagement, with a particular focus on the less-understood, *health* civic engagement, or civic engagement in health-related and health-specific activities. Using data from a nationally representative sample of adults in the United States (U.S.), we examined whether the extent to which individuals feel they belong in their community (i.e., perceived sense of community) and the value they placed on investing in community health were associated with individuals’ health civic engagement.

**Methods:**

Using data collected on 7187 nationally representative respondents from the 2018 National Survey of Health Attitudes, we examined associations between sense of community, valued investment in community health, and perceived barriers to taking action to invest in community health, with health civic engagement. We constructed continuous scales for each of these constructs and employed multiple linear regressions adjusting for multiple covariates including U.S. region and city size of residence, educational attainment, family income, race/ethnicity, household size, employment status, and years living in the community.

**Results:**

Participants who endorsed (i.e., responded with mostly or completely) all 16 sense of community scale items endorsed an average of 22.8% (95%CI: 19.8–25.7%) more of the health civic engagement scale items compared with respondents who did not endorse any of the sense of community items. Those who endorsed (responded that it was an important or top priority) all items capturing valued investment in community health endorsed 14.0% (95%CI: 11.2–16.8%) more of the health civic engagement items than those who did not endorse any valued investment in community health items.

**Conclusions:**

Health civic engagement, including voting and volunteering to ultimately guide government decisions about health issues, may help improve conditions that influence health and well-being for all. Focusing on individuals’ sense of community and highlighting investments in community health may concurrently be associated with increased health civic engagement and improved community and population health.

## Background

Evidence on the role of socioeconomic and environmental determinants in shaping individual and community health has been building for many years [1–3], and it is increasingly understood that addressing the structural drivers of health outcomes requires broad-based efforts that reach beyond the traditional health care and public health systems. This includes significant reallocations or realignment of resources [4], and changes in laws, policies, and regulations [5]. In democratic systems, civic engagement – through formal voting, advocacy, and involvement in civic organizations – is one way in which people ensure that governmental and nongovernmental actors work to change laws and policies or take other actions that promote healthy communities. In addition to providing a potential avenue for system change, civic engagement has been shown to be positively associated with physical health [6–10], health behaviors [11, 12], mental health [8, 12, 13], and well-being [14, 15]. The specific impact may vary across specific health conditions [16, 17]. Nonetheless, these associations hold across various forms of civic engagement, including voting, membership in community organizations, and direct community service [18].

The role of civic engagement in both systems-level and individual health is reflected, among other places, in the Culture of Health action framework advanced by the Robert Wood Johnson Foundation [19, 20], which is founded on a vision in which “everyone in our diverse society leads healthier lives now and for generations to come” and is premised that accelerating health improvement requires broader collective action and cultural and social mobilization. The Culture of Health action framework [19] identifies civic engagement as a key driver needed to *make a health a shared value*, which is one of the framework’s four action areas. Civic engagement is viewed as a critical process through which “people develop and use knowledge, skills and voice to cultivate positive change,” and translate the value they place on community health into action. According to the framework, civic engagement is linked to two additional drivers that help support and conduct civic engagement [18].

One of those drivers is *mindset and expectations*, or the way in which individuals and communities frame the importance of health and well-being, the factors that influence it, and the shared role of individuals and organizations in actively promoting health and well-being [21]. Indeed, previous research in political science suggests that individuals are more willing to engage civically to promote public goods that they place a high value on [22, 23]. Moreover, other research suggests that how individuals understand and frame the causes of and degree of personal responsibility for health conditions such as diabetes (e.g., as the result of individual behavior vs. environmental conditions) is linked to their willingness to support investments in community health [16, 24, 25]. The importance of mindset and expectations in health is also supported by research and practice evidence from social network theory, community resilience, narrative theory, well-being science, and asset-based community development, each field articulating pathways by which individual and community sentiment about an issue is formed and addressed [21, 26, 27].

The other driver is *sense of community*. This driver captures an individual’s sense that he or she has a personal connection to the community (membership), that the community matters to him or her (belonging), and he/she has shared experiences with others (events, memories) in the community. Sense of community is positively associated with personal well-being and is framed as another key driver of *making health a shared value* [26]. Sense of community has been shown to be significantly and positively associated with community and civic participation in adults [28–31].

To date, the literature linking civic engagement, mindset and expectations, and sense of community remains somewhat limited, and there has not been much examination of the link between these two drivers and civic engagement specifically related to health activities. There has also been limited work around the relationship of health civic engagement to other factors including perceived barriers to taking action to invest in community health, and perception of how external organizations and agencies may have an impact on community health. Given that the Culture of Health action framework also builds on the idea that both individual and community action is needed to improve health, well-being, and health equity, understanding the public’s perceptions about readiness to engage individually and collectively is key. To illuminate those relationships, our team used the 2018 National Survey of Health Attitudes fielded by Robert Wood Johnson Foundation (RWJF) and RAND to address two aims: first, to describe patterns in health civic engagement in the U.S. population and, second, to explore the relationship between health civic engagement, sense of community, valued investment in community health, perceived barriers to taking action to invest in community health, and the perception of the role of external organizations and agencies in community health, net of other social and demographic factors known to be related to civic engagement and potentially to health civic engagement.

## Methods

We build upon a new and innovative data set on health attitudes in the U.S. to cross-sectionally analyze factors that account for variations in health civic engagement. As part of a larger effort to track progress in building a Culture of Health [18, 19], RAND worked with RWJF to design and field the National Survey of Health Attitudes, aimed to provide insight on how people in the U.S. think about, value, and prioritize health and consider issues of health and health equity. The survey was initially fielded in 2015, and an updated version was fielded in 2018. The later version serves as the focus of this study with more detail provided elsewhere [32, 33]. Both versions were administered online and survey data for 2018 was collected from 7187 respondents via the RAND American Life Panel (ALP) and the Ipsos KnowledgePanel. The sample is based on nationally representative internet panels whose members are recruited via probability-based sampling methods. A central goal of the survey was to measure *making health a shared value*, within the Culture of Health action framework. As such, the survey included questions about how the public views the value of investing in community health, the perceived sense of community, and health civic engagement. The survey also contained other items including measurement of barriers to taking action to invest in community health as well as perceptions of what has an impact on community health.

Health civic engagement was measured by a set of questions that asked about activities in which individuals are involved that might influence government and civil society actions about health, including two questions about voting for a candidate based on his/her position, and questions about contribution of time or money to organizations working to pass health laws or policies, lobbying for a health-related causes, and attending meetings or working with neighbors to fix community problems (Table 1). Sense of community, in turn, was measured via a set of 16 items (Full Sense of Community Scale) including those from the Sense of Community Index (SCI) [36], which consists of two subscales – membership (six items) and emotional connection (six items) and four additional items that were specifically designed for this survey to capture health-related sense of community and community capacity to improve health (Table 1).

**Table 1:** Operationalization of Measures

Concept	Measure Items
Health civic engagement	There are many activities that a person could do to influence government decisions about health issues. During the past year have you. . . A. Voted for or against a candidate for public office because of his/her position on a health problem or issue B. Voted for or against a candidate for public office because of his/her position on other issues such as education, public safety, or community funding C. Contributed time or money to an organization working to pass a health law or policy at the local, state or national level D. Lobbied or advocated for a health-related cause in your community. (This may include signing a petition, calling a public official, disseminating information via social media, participating in demonstrations) E. Attended a civic meeting or worked with neighbors to fix community problems *SOURCES: America’s Health Agenda: Priorities and Performance Rating Survey* [34] *(revised), and CPS Civic Engagement Supplement* [35]*; adapted by RAND and RWJF.*
Sense of Community	The following statements about community refer to your neighborhood. How well do each of the following statements represent how you feel about this community?—not at all, somewhat, mostly, or completely
Membership	A. I can trust people in this community B. I can recognize most of the members of this community C. Most community members know me D. This community has symbols and expressions of membership such as clothes, signs, art, architecture, logos, landmarks, and flags that people can recognize E. I put a lot of time and effort into being part of this community F. Being a member of this community is part of my identity
Emotional	G. It is very important to me to be a part of this community H. I am with other community members a lot and enjoy being with them I. I expect to be a part of this community for a long time J. Members of this community have shared important events together, such as holidays, celebrations, or disasters K. I feel hopeful about the future of this community L. Members of this community care about each other *SOURCE: Sense of Community Index* [36]
Health-related	M. My community can work together to improve its health N. My community has the resources to improve its health O. My community works together to make positive change for health P. I know my neighbors will help me stay healthy *SOURCE: RAND*
Valued Investment in Community Health	Should the following be a top priority, important but not a top priority, or not a priority at all for communities for the following: A. Making sure that the disadvantaged have an equal opportunity to be healthy; B. Making sure that healthy foods are for sale at affordable prices in communities where they are not; C. Making sure that there are safe, outdoor places to walk and be physically active in communities where there are not any; D. Making sure that there is decent housing available for everyone who needs it; *SOURCE: American Health Values Segmentation Study* [37] E. Making sure that there are bike lanes, sidewalks for walking, and public transportation available so that people do not have to always rely on cars. *Or* Making sure that there is public transportation, sidewalks or walking, and bike lanes available so that people do not have to always rely on cars.^a^ *SOURCE: RAND* F. Do you agree or disagree with the following statement? “It is the obligation of the government to ensure that everyone has access to health care as a fundamental right.” *SOURCE: RAND & RWJF.*
Barriers to taking action to invest in community health	Whether or not you have taken action to improve health in your community, many people face barriers to getting involved. Thinking about the following statements, please rate the extent to which you think this has been a barrier for people in your community. A. People don’t know how to get involved or where to start B. People don’t think their involvement will really make a difference in changing the health of the community C. People offer suggestions but only those coming from certain groups or individuals are addressed D. There are other issues people care more about *SOURCES: RAND and RWJF*

^a^Respondents were randomly assigned to receive either the first or second wording of this question. Respondents were 3 percentage points more likely to endorse this statement as a top priority when public transportation was listed first than when bike lanes were listed first

Valued investment in community health was captured with five questions about how individuals prioritize the importance of health opportunity and access via greater investment in improving access to healthy and affordable food, safe spaces for outdoor activities, safe housing, and different modes of transportation (Table 1). Because individuals may be less likely to engage civically when they perceive significant barriers to doing so [23, 38, 39], we also examined barriers to taking action to invest in community health. This included items eliciting perceptions about whether individuals know how to get involved, whether that involvement will make a difference, and whether they think that meaningful influence over community health is only limited to certain community groups.

Other variables we considered included self-rated health [40], financial problems because of or experience with a chronic health condition, and burden of caring for others. Years living in the community provided a proxy for the rootedness in a community. Several decades of research suggest that willingness to vote or undertake other forms of civic engagement is associated with multiple socioeconomic factors [41, 42], thus we included measures of employment status, educational attainment, marital status, race/ethnicity and city size of residence as multiple socioeconomic and demographic variables that could be related to health civic engagement. Finally, age and gender were included as covariates given prior work showing the influence of both age and gender to civic engagement [43].. The main measures used in our analysis are listed in Table 1.

### Statistical analysis

We used data from 7187 survey respondents to examine civic engagement, sense of community, and valued investment in community health. We first identified the groups of survey items that comprised each of these constructs, and assessed scale reliability for each set using Cronbach’s alpha; the alpha statistic was 0.93 for the full sense of community items, and 0.83, 0.85, and 0.78 for the membership, emotional connection, and health subscale items respectively. The alpha was 0.74 for our full set of health civic engagement items, 0.67 for valued investment in community health, and 0.77 for barriers to taking action to invest in community health. We conducted exploratory factor analyses to generate continuous factors for each construct. However, for ease of interpretation, we generated a simple summary value for each construct. This was calculated by the proportion of items in the domain with which a respondent either mostly or completely agreed, or in the case of the priority questions, rated as important or a top priority. This resulted in a summary proportion between 0 and 1. Maintaining the scale between 0 and 1 allows simple interpretation of change, where a 1 unit increase represents a change from endorsing no items to endorsing all items in the domain.

To estimate the extent to which sense of community and investment in community health was associated with civic engagement, we fit linear regression models of the civic engagement outcome, with sense of community and investment in community health as predictors of interest, with responses weighted to account for the survey sampling design. We fit models first for unadjusted estimates, and then adjusted for age, race/ethnicity, sex, marital status, geographic region (Northeast, Midwest, South, West), education, family income, household size, employment, length of time in the community, and rurality. We also included an age-squared term to allow for a potential non-linear relationship between age and health civic engagement. We limited modeling to those subjects with complete data, which comprised 96% of the total sample.

We additionally explored the inclusion of interaction terms in the models that would allow us to assess whether relationships between sense of community and health civic engagement may differ by race/ethnicity or geographic region. All data management, factor analyses, and regression modeling were conducted using Stata software, version 15.

## Results

### Respondent characteristics

Table 2 shows respondent characteristics of the National Survey of Health Attitudes. We observed a fairly even age distribution, with most respondents between 18 and 69. Approximately 13% of respondents were 70 or older. Respondents were 52% female, 61% were married or living with a partner, and household income was fairly evenly distributed between respondents who reported a household income of less than $30,000 per year and those who reported an income of $100,000 per year or higher. Forty percent of respondents had up to a high school education; 28% completed some college, and 32% had completed college or higher. Approximately 70% of respondents had between two and five people living in the household.

**Table 2:** National Survey of Health Attitudes Participants

	Observed frequency (unweighted) *N* = 7187	Survey weighted percent
**Age in years**		
18 to 29	657	20.0%
30 to 39	934	17.4%
40 to 49	1092	16.3%
50 to 59	1579	17.9%
60 to 69	1671	15.9%
70 to 79	976	9.7%
80 or older	278	2.8%
**Sex**		
Male	3317	48.2%
Female	3870	51.8%
**Marital status**		
Married or living with a partner	4605	61.1%
Separated, Divorced or Widowed	1395	15.6%
Never married	1187	23.4%
**Household income category**		
Less than $30 K	1384	23.4%
$30 K to $60 K	1824	27.3%
$60 K to $100 K	1665	23.2%
$100 K or higher	2307	26.1%
**Number living in household**		
One person	1495	14.8%
Two people	2872	34.4%
Three or four	2034	35.8%
Five or more	786	15.1%
**Geographic region**		
Northeast	1317	17.9%
Midwest	1487	20.2%
South	2564	36.9%
West	1781	25.1%
**Education category**		
Up to High School	2019	40.1%
Some College	2225	28.4%
Bachelor’s degree or higher	2943	31.5%
**Employment**		
Employed (working as a paid employee, or self-employed)	4147	60.1%
Retired	1885	19.1%
Unemployed (looking for work, on temporary layoff, disabled, or other)	1155	20.8%
**Time living in the community**		
Less than 5 years	1391	22.6%
5 to 9 years	972	15.1%
10 to 19 years	1713	24.0%
20 or more years	3027	38.3%
**City size**		
Not a Town	971	13.9%
Small Town (2.5-50 K)	749	10.4%
Midsized City (50-500 K)	1493	21.5%
Large City (500 K+)	3945	54.2%
**Self-rated health**		
Excellent	740	10.4%
Very good	2796	37.3%
Good	2475	36.3%
Fair	874	12.9%
Poor	219	3.2%
**Poor health of another affects life**		
No	4127	61.6%
Yes	2965	38.4%
**Chronic health condition**		
No	4417	64.3%
Yes	2654	35.7%
**Financial problems due to health**		
No	5335	74.7%
Yes	1744	25.3%
**How often care for ailing others**		
More than once per week	1382	19.1%
Up to once a week	2364	30.7%
Never	3341	50.2%

Source: RAND

Respondents were from the Northeast, Midwest, South and West, with the highest percentage of respondents (37%) from the South. Sixty percent of respondents were employed and 38% of respondents had spent 20 or more years living in their community. Fifty-four percent of respondents lived in a large city (500,000+ residents) and 84% rated their health as good, very good, or excellent. About 38% of respondents reported that poor health of another affected their life and 36% reported that they suffered from a chronic health condition. Approximately 25% reported a financial problem due to health. Nearly 50% of respondents reported that they cared for others at least once per week who were ailing.

### Health civic engagement, sense of community, valued Investment in Community Health, and barriers to action

Our first aim was to describe key patterns in health civic engagement. Table 3 shows the breakdown of respondent answers specific to each item that comprised health civic engagement, sense of community, valued investment in community health and participants report of barriers to acting to improve community health as listed in Table 1. Within health civic engagement, it is notable that 51% of participants reported that they voted for or against a candidate for public office because of his/her position on issues such as education, public safety or community funding, which are upstream drivers of health but not directly health specific. On the other hand, 19% reported contributing time or money to an organization working to pass a health law or policy, and about 21% reported lobbying or advocating for a health-specific cause in their community. About 22% reported engagement through attendance at a civic meeting or working with neighbors to fix community problems.

**Table 3:** Main constructs

	Observed frequency (unweighted) *N* = 7187	Survey Weighted Percentage
**Health Civic Engagement**		*Percent who have done activity to influence decisions about health issues during the past year*
A. Voted for or against a candidate for public office because of his/her position on a health problem or issue	3235	41.12%
B. Voted for or against a candidate for public office because of his/her position on other issues such as education, public safety, or community funding	4075	51.89%
C. Contributed time or money to an organization working to pass a health law or policy at the local, state or national level	1442	18.64%
D. Lobbied or advocated for a health-related cause in your community (may include signing a petition, calling a public official, disseminating information via social media, participating in demonstrations)	1617	21.15%
E. Attended a civic meeting or worked with neighbors to fix community problems	1792	21.72%
**Sense of Community**		*Percent who respond mostly or completely*
***Membership Subscale (A-F)***		
A. I can trust people in this community	3440	45.00%
B. I can recognize most of the members of this community	2020	28.76%
C. Most community members know me	1746	24.38%
D. This community has symbols and expressions of membership such as clothes, signs, art, architecture, logos, landmarks, and flags that people can recognize	1977	27.74%
E. I put a lot of time and effort into being part of this community	1673	22.64%
F. Being a member of this community is part of my identity	1950	26.94%
***Emotional Connection Subscale (G-L)***		
G. It is very important to me to be part of this community	2804	37.80%
H. I am with other community members a lot and enjoy being with them	1701	23.34%
I. I expect to be a part of this community for a long time	3912	51.58%
J. Members of this community have shared important events together, such as holidays, celebrations, or disasters	2618	35.63%
K. I feel hopeful about the future of this community	3817	49.81%
L. Members of this community care about each other	3063	41.08%
***Health Subscale (M-P)***		
M. My community can work together to improve its health	2742	38.17%
N. My community has the resources to improve its health	3102	41.57%
O. My community works together to make positive change for health	1843	25.76%
P. I know my neighbors will help me stay healthy	*1316*	*19.08%*
**Valued Investment in Community Health**		*Percent who think these things are a top priority for communities*
A. Making sure that the disadvantaged have equal opportunity to be healthy	3200	44.94%
B. Making sure that healthy foods are for sale at affordable prices in communities where they are not	3054	44.75%
C. Making sure that there are safe, outdoor places to walk and be physically active in communities where there aren’t any	2453	36.54%
D. Making sure that there is decent housing available for everyone who needs it	3094	44.40%
E. Making sure that there are bike lanes, sidewalks for walking and public transportation available so that people do not have to always rely on cars	1543	23.43%
F. It is the obligation of the government to ensure that everyone has access to health care as a fundamental right.	2261	32.25%
**Barriers to Acting to Improve Community Health**		*Percent who think these things are a major barrier to getting involved to improve health in their community*
A. People don’t know how to get involved or where to start	1186	18.01%
B. People don’t think their involvement will really make a difference in changing the health of the community	1555	23.30%
C. People offer suggestions but only those coming from certain groups or individuals are addressed	1039	15.43%
D. There are other issues people care more about	1304	19.27%

Items within sense of community were broken into three subscales: membership, emotional connection and health. Within the membership subscale, 45% of respondents said they felt they could trust people in their community, 29% reported being able to recognize most of the members of their community, and about 27% reported that being a member of their community was part of their identity. Respondents’ emotional connection varied: 38% reported that it was very important to be part of their community and 52% said they expected to be part of the community for a long time. Nearly 50% felt hopeful about the future of their community and 41% said members of their community cared about each other. Health subscale questions asked about the community working together and having resources to improve its health. Thirty-eight percent reported that their community can work together to improve its health and only 19% said that they knew their neighbors would help them stay healthy. This is particularly notable given that Culture of Health action Framework is partially premised on advancing community action to improve health.

Finally, as far as valued investment in community health, 45% of respondents reported their top priorities were to make sure that the disadvantaged had equal opportunity to be healthy, decent housing was available for all who needed it, and healthy foods were available at affordable prices in communities. Just 23% of respondents thought there should be bike lanes, sidewalks for walking and public transportation so that people did not need to rely on cars. Respondents thought that barriers to taking action to invest in community health included people not thinking their involvement would really make a difference in changing the health of the community (23%) and that there were other issues (not specified) that people cared more about (19%).

### Predictors of health civic engagement

Our second aim was to explore the relationship between health civic engagement and individuals’ reports on sense of community, valued investment in community health, and perceived barriers to engagement, net of other factors known to be related to health-specific civic engagement. Results of this analysis are shown in the regression analyses in Table 4. Model 1 shows the full set of items related to sense of community, and models 2, 3 and 4 show the sense of community subscales of membership, emotional connection and health. Each of the models show qualitatively similar results, independent of which sense of community subscale (or full scale) was used. We observed that even after adjusting for all other covariates, sense of community, regardless of whether it is conceptualized as the full set of questions, or as one of the three subscales (membership, emotional connection and health), has a strong statistically significant (*p* < .0001) positive association with health civic engagement. As sense of community increased, so did health civic engagement. Specifically, respondents who endorsed all items on the full sense of community scale endorsed an average of 22.8% (95%CI: 19.8–25.7%) more of the health civic engagement items than those who did not endorse any sense of community items. Those who endorsed all the items in the sense of community membership subscale endorsed an average of 21.1% (95% CI: 18.3, 23.8%) more of the health civic engagement items than those not endorsing any of the membership items. The analogous difference in health civic engagement endorsement for the emotional connection subscale was 17.5% (95% CI: 15.1, 20.0%) and 15.8% (95% CI: 13.3, 18.4%) for the health subscale.

**Table 4:** Regression estimates of relationships between full health civic engagement scale and the full sense of community scale and its subscales

	Full Sense of Community Scale			Membership Subscale			Emotional Connection Subscale			Health Subscale		
	β	se	***p***-value	β	se	***p***-value	β	se	***p***-value	β	se	***p***-value
Sense of Community												
Full Set of Sense of Community Items	**0.228**	**0.015**	**0.000**									
Membership subscale				**0.211**	**0.014**	**0.000**						
Emotional connection subscale							**0.175**	**0.013**	**0.000**			
Health subscale										**0.158**	**0.013**	**0.000**
Value Invested in Community Health	**0.140**	**0.014**	**0.000**	**0.146**	**0.014**	**0.000**	**0.143**	**0.014**	**0.000**	**0.143**	**0.014**	**0.000**
Barriers to Acting to Improve Community Health	0.000	0.015	0.976	−0.003	0.015	0.866	−0.001	0.015	0.958	0.000	0.015	0.988
Age in years												
Linear term	0.003	0.001	0.021	0.003	0.001	0.029	**0.003**	**0.001**	**0.043**	**0.003**	**0.001**	**0.023**
Quadratic term	0.000	0.000	0.533	0.000	0.000	0.680	0.000	0.000	0.661	0.000	0.000	0.637
Race/Ethnicity			**0.001**			**0.001**			**0.001**			**0.002**
Non-Hispanic White (reference)	0.000	–	–	0.000	–	–	0.000	–	–	0.000	–	–
Non-Hispanic Black	**0.044**	**0.015**	**0.003**	**0.041**	**0.015**	**0.005**	**0.047**	**0.015**	**0.002**	**0.044**	**0.015**	**0.003**
Hispanic	0.007	0.013	0.569	0.005	0.013	0.671	0.008	0.013	0.553	0.012	0.013	0.352
Non-Hispanic Asian or Pacific Islander	**−0.050**	**0.024**	**0.035**	**−0.053**	**0.024**	**0.025**	− 0.045	0.024	0.058	**−0.049**	**0.024**	**0.041**
Non-Hispanic All other races	**0.055**	**0.024**	**0.020**	**0.054**	**0.024**	**0.025**	**0.059**	**0.024**	**0.012**	**0.049**	**0.023**	**0.038**
Female	−0.018	0.008	0.019	−0.017	0.008	0.036	−0.021	0.008	0.007	−0.018	0.008	0.023
Marital status			**0.536**			**0.501**			**0.637**			**0.553**
Married or living with partner	−0.011	0.013	0.370	−0.009	0.013	0.477	−0.010	0.013	0.414	−0.009	0.013	0.471
Sep., Div., or Wid.	−0.016	0.014	0.269	−0.017	0.014	0.244	−0.013	0.014	0.358	−0.016	0.015	0.277
Never married (reference)	0.000	–	–	0.000	–	–	0.000	–	–	0.000	–	–
Region			**0.000**			**0.000**			**0.000**			**0.000**
Northeast	0.006	0.011	0.612	0.005	0.011	0.654	0.006	0.011	0.575	0.003	0.011	0.755
Midwest	−0.007	0.011	0.532	− 0.002	0.011	0.845	−0.007	0.011	0.508	−0.008	0.011	0.438
South (reference)	0.000	–	–	0.000	–	–	0.000	–	–	0.000	–	–
**West**	**0.051**	**0.010**	**0.000**	**0.053**	**0.010**	**0.000**	**0.050**	**0.010**	**0.000**	**0.049**	**0.010**	**0.000**
Education			**0.000**			**0.000**			**0.000**			**0.000**
Up to High school (reference)	0.000	–	–	0.000	–	–	0.000	–	–	0.000	–	–
Some College	**0.066**	**0.010**	**0.000**	**0.067**	**0.010**	**0.000**	**0.066**	**0.010**	**0.000**	**0.066**	**0.010**	**0.000**
BA or higher	**0.129**	**0.011**	**0.000**	**0.132**	**0.011**	**0.000**	**0.130**	**0.011**	**0.000**	**0.132**	**0.011**	**0.000**
Family Income (in units of $10 K)	**0.003**	**0.001**	**0.000**	**0.004**	**0.001**	**0.000**	**0.003**	**0.001**	**0.000**	**0.003**	**0.001**	**0.000**
Household size (number of individuals)	−0.004	0.003	0.163	−0.004	0.003	0.180	− 0.004	0.003	0.153	−0.004	0.003	0.181
Employed	0.010	0.009	0.285	0.010	0.009	0.279	0.010	0.009	0.270	0.010	0.009	0.307
Years living in community			**0.002**			**0.004**			**0.001**			**0.000**
Less than 5 years (reference)	0.000	–	–	0.000	–	–	0.000	–	–	0.000	–	–
5 to 9 years	**0.038**	**0.013**	**0.004**	**0.037**	**0.013**	**0.006**	**0.041**	**0.013**	**0.002**	**0.040**	**0.013**	**0.003**
10 to 19 years	**0.041**	**0.012**	**0.000**	**0.039**	**0.012**	**0.001**	**0.044**	**0.012**	**0.000**	**0.046**	**0.012**	**0.000**
20 or more years	**0.036**	**0.011**	**0.001**	**0.034**	**0.011**	**0.003**	**0.039**	**0.011**	**0.001**	**0.045**	**0.011**	**0.000**
City size			**0.030**			**0.029**			**0.026**			**0.066**
Not a Town	−0.012	0.011	0.269	−0.017	0.011	0.134	−0.014	0.011	0.202	−0.001	0.011	0.945
Small Town (2.5 - 50 K)	**−0.034**	**0.013**	**0.008**	**−0.034**	**0.013**	**0.008**	**−0.036**	**0.013**	**0.006**	**−0.028**	**0.013**	**0.029**
Midsized City (50 - 500 K)	**−0.020**	**0.010**	**0.046**	**−0.020**	**0.010**	**0.050**	−0.019	0.010	0.057	−0.018	0.010	0.066
Large City (500 K+) (reference)	0.000	–	–	0.000	–	–	0.000	–	–	0.000	–	–
Self-rated health			**0.005**			**0.001**			**0.002**			**0.001**
Excellent	**0.046**	**0.016**	**0.005**	**0.050**	**0.016**	**0.002**	**0.051**	**0.016**	**0.002**	**0.053**	**0.016**	**0.001**
Very good	0.006	0.009	0.519	0.008	0.009	0.357	0.008	0.009	0.372	0.009	0.009	0.315
Good (reference)	0.000	–	–	0.000	–	–	0.000	–	–	0.000	–	–
Fair	−0.023	0.012	0.064	**−0.026**	**0.012**	**0.034**	−0.022	0.012	0.072	**−0.026**	**0.012**	**0.034**
Poor	−0.032	0.022	0.144	−0.031	0.022	0.164	−0.034	0.022	0.129	−0.037	0.023	0.102
Poor health of another impacts life	**0.053**	**0.009**	**0.000**	**0.053**	**0.009**	**0.000**	**0.052**	**0.009**	**0.000**	**0.054**	**0.009**	**0.000**
Chronic health condition	0.009	0.009	0.316	0.010	0.009	0.265	0.009	0.009	0.322	0.009	0.009	0.307
Financial problems due to health	**0.068**	**0.010**	**0.000**	**0.067**	**0.010**	**0.000**	**0.068**	**0.010**	**0.000**	**0.066**	**0.010**	**0.000**
Helps others who are ailing			**0.000**			**0.000**			**0.000**			**0.000**
More than once per week	**0.075**	**0.012**	**0.000**	**0.073**	**0.012**	**0.000**	**0.079**	**0.012**	**0.000**	**0.084**	**0.012**	**0.000**
Up to once a week	**0.073**	**0.009**	**0.000**	**0.073**	**0.009**	**0.000**	**0.078**	**0.009**	**0.000**	**0.080**	**0.009**	**0.000**
Never (reference)	0.000	–	–	0.000	–	–	0.000	–	–	0.000	–	–

Similarly, respondents who valued investment in community health were also more likely to participate civically. Those who endorsed all items around valuing investment in community health endorsed 14% (95% CI: 11.2, 16.8%) more of the health civic engagement items than those who did not endorse valuing investment in community health. This too was a strong, statistically significant (*p* < .0001) positive relationship. Endorsement of items related to barriers to action to improve community health, including whether respondents thought that external groups (community members, businesses, government) could influence community health, or whether respondents believed that there were barriers to taking action to invest in community health, did not show a statistically significant relationship with health civic engagement. We note that these results were qualitatively similar, in both direction and statistical significance, to those we generated using exploratory factor analyses to derive the construct scales (data not shown).

Covariates that were positively associated with health civic engagement include rating one’s health as excellent (compared with ‘good’), responding that the poor health of another has impacted one’s life, having financial problems due to health, and having helped (or helping) others who are ailing. We observed relationships with educational attainment, race/ethnicity, and city size of residence as well. As Table 4 indicates, we found that non-Hispanic blacks have a significant positive association with health civic engagement, compared with non-Hispanic whites. Non-Hispanic Asians were less likely to engage civically. Compared with living in the South, living in the West was strongly positively associated with health civic engagement. Higher education is positively associated with higher levels of health civic engagement.

**Fig. 1:**
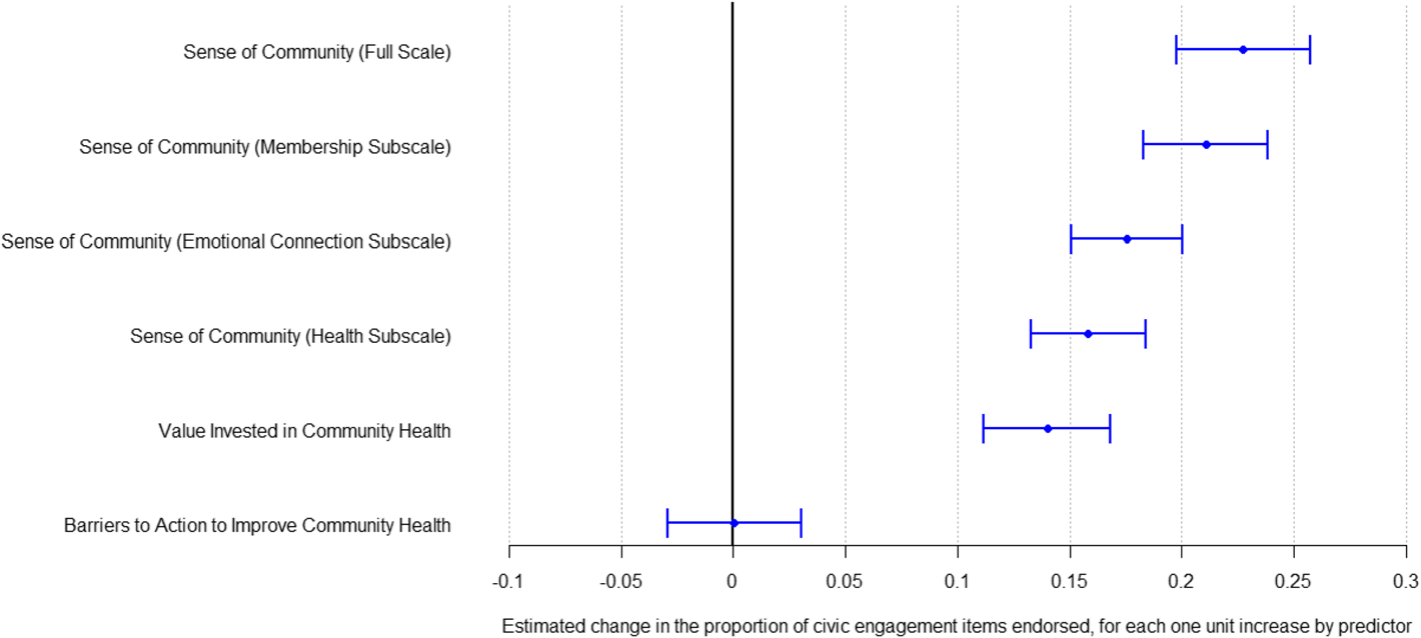
Sense of Community, Valued Investment in Community Health, and Barriers to Action to Improve Community Health as Predictors of Health Civic Engagement

Figure 1 shows the fully adjusted associations of our main outcomes of health civic engagement with our main variables of interest: each of the sense of community subscales, value invested in community health, the impact of external groups on community health, and barriers to taking action to invest in community health. In sum, one’s sense of community to a large extent, and the value invested in community health to a lesser extent, are positively and significantly associated with health civic engagement. One’s perception of barriers to action to improve community health is not significantly associated with health civic engagement.

## Discussion and public health implications

This analysis set out to explore patterns in health civic engagement, and especially the relationship with sense of community and mindset and expectations (identified as key drivers of *making health a shared value* in the Culture of Health Action Framework), net of other factors known to be related to health civic engagement.

As expected, respondents reporting a strong sense of community and those who prioritize community investments in health more frequently reported engaging civically around health, controlling for variations in measures of socioeconomic status, perceived barriers, and a host of other variables. This is consistent with the view, embodied in the Culture of Health action framework and grounded in the literature, that individuals who view health as an issue requiring collective or community investment and who identify strongly with their communities are more likely to engage civically specifically to address health issues [21, 44, 45].

Our findings also confirm that, on a population level, overall health-related sense of community and health civic engagement are generally limited. Given that building a Culture of Health in America requires some amount of community action, the fact that less than 40 % of respondents felt their community could work together to improve health suggests room for further work and action. Action could include efforts that bring communities together and/or initiatives and programs to promote residents to be more involved with decisions surrounding their communities’ futures. More institutional supports could be imagined with improved and increased civics included in primary education. In addition, with one exception (voting on an issue related to education, public safety or another community issue), the health civic engagement items did not exceed half of the respondents, and the case of advocating specifically for “health policy” was reported positively by just one-fifth of respondents. This may suggest that an entry point to activate health-specific action is through these *health-related* activities on upstream drivers such as education or public safety.

The findings also have potentially interesting implications for how we understand the barriers to health civic engagement. We did not find clear evidence that those perceiving barriers to act to improve community health (e.g., not knowing how to get involved, not believing that involvement makes a difference) were less likely to engage civically around health, suggesting that addressing these attitudinal barriers may not be enough to activate health civic engagement. The limited literature in this space to support or contradict our finding highlights the question of how to inspire and motivate health civic engagement.

We did find evidence that one’s own poor health, was associated with lower levels of civic engagement around health. This aligns with a number of earlier findings [18] from the U.S. and elsewhere suggesting that those in poor health or with disabilities are less likely to vote or engage civically in other ways. In one study, people with disabilities were 20 percentage points less likely to vote than people without disabilities of similar demographics [9]. In another longitudinal study in Ireland and Britain, individuals with poor self-rated health were significantly less likely to report voting in past general elections [46]. While the direction of the causality is not always clear, it might point to the need to broaden access to mail-in ballots and other measures to lower the burdens of political participation. It also suggests that activities to expand health civic engagement may need to address the unique challenges faced by those with chronic health conditions, including, for example, difficulty with mobility and regular participation in activities which require travel. Respondents who reported that poor health of a friend of family member impacts their life, those who reported financial problems due to health, and those who reported helping others who were ailing were all more likely to be civically engaged in health-related activity. This suggests that some degree of experiencing a health-related challenge or barrier may motivate individuals to take action. Without being able to assess the direction of causality, this does suggest the possibility that the burdens of caring for others may be a catalyst for health civic engagement, or perhaps that those who help others in need are also more inclined towards involvement in the community.

Finally, consistent with many other studies of civic engagement and political participation, we found that those with higher levels of education and higher income were more likely to engage civically around health. In light of previous research cited above linking civic engagement with individual health [16, 47, 48] benefits, such findings are an important reminder that the health and well-being benefits of civic engagement do not accrue equitably to all. First, as efforts are underway to advance health equity, these findings further underscore the challenges in engaging Americans in health civic engagement. Second, the health benefits of civic engagement are also not experienced equally. We did observe a notable finding worthy of further investigation: African American/black respondents were more likely to engage on health issues civically than white Americans, while Asian respondents were less likely than all other groups, adjusting for all other factors. There are many potential explanations for this disparity (data not shown), given that in the broader survey, we also observe the same racial/ethnic differences in willingness to invest in community health (only 17% of African Americans/blacks would not invest in any community health priorities vs. 33% of white Americans). It could be that African American/blacks believe there is a greater need to engage civically for health, or there may be a stronger view that this approach to health improvement will be more effective.

This study offers important new insights about health civic engagement and the role of community health attitudes and sense of community. Still, the findings are subject to a few limitations. First, the cross-sectional nature of the data used in this analysis means we cannot assume the observed relationships between civic engagement, sense of community and value placed in community health investments are causal. Perhaps more importantly, though, the study was unable to assess the extent to which individual civic engagement impacts the health of communities through fostering changes in socio-economic drivers of health, or the possibility that civic engagement may promote policies or other collective actions that compromise the health of some groups (e.g., lobby for weakening of environmental protections). This also reflects a broader tendency in the current research (e.g., 43, 44) to focus on health impacts on those who are engaging in civic activities [19].

## Conclusion

Civic engagement is a potential avenue through which individuals can take actions to make their communities more conducive to health, and also is linked to improvements in the health of civically engaged individuals. This paper presented a unique analysis of health civic engagement. To date, there has been little research on whether individual levels of civic engagement are linked to improvements in community health and whether there are differences related to health civic engagement. Our analysis of a nationally representative survey suggests that civically engaged individuals are more likely to have a strong sense of community and to view community health as a priority. This is consistent with the Culture of Health action framework, which outlines civic engagement, sense of community, and mindset and expectations around community health as key drivers in *making health a shared value*. While preliminary in nature, this research points to the potential importance of civic engagement as a means for improving community health. Future research should seek to use longitudinal data to better understand the life-trajectories and contextual factors that lead to high levels of health civic engagement, to better understand the direction of causality – i.e., whether civic engagement promotes better health or vice-versa – and explore the implications of civic engagement for community health and health equity.

## Data Availability

Data are currently being reviewed at ICPSR, and can be search for under “the National Survey of Health Attitudes” at the website https://www.icpsr.umich.edu/web/pages/HMCA/archive.html Or alternatively, please contact either Dr. Katherine Carman (kcarman@rand.org) or Dr. Anita Chanda (chandra@rand.org).

## References

[1] Braveman P, Egerter S, Williams DR (2011). The social determinants of health: coming of age. Annu Rev Public Health.

[2] Marmot M, Wilkinson R (1999). Social determinants of health.

[3] Smedley BD, Syme SL (2001). Committee on capitalizing on social S, behavioral research to improve the Public's H. promoting health: intervention strategies from social and behavioral research. Am J Health Promot.

[4] Bradley E, Taylor L (2013). The American health care paradox: why spending more is getting us less.

[5] Carey G, Crammond B (2015). Action on the social determinants of health: views from inside the policy process. Soc Sci Med.

[6] Buck-McFadyen E, Akhtar-Danesh N, Isaacs S, Leipert B, Strachan P, Valaitis R (2018). Social capital and self-rated health: a cross-sectional study of the general social survey data comparing rural and urban adults in Ontario. Health Soc Care Community.

[7] Danso K (2017). Immigrant health disparities: does neighborliness improve health?. J Sociol Soc Welf.

[8] Ojeda C, Pacheco J. Health and voting in young adulthood. Br J Polit Sci. 2019;49(3):1163–1186.

[9] Schur L, Shields T, Kruse D, Schriner K (2002). Enabling democracy: disability and voter turnout. Polit Res Q.

[10] Söderlund P, Rapeli L (2015). In sickness and in health: personal health and political participation in the Nordic countries. Politics Life Sci.

[11] Albright K, Hood N, Ma M, Levinson AH (2015). Smoking and (not) voting: the negative relationship between a health-risk behavior and political participation in Colorado. Nicotine Tob Res.

[12] Pillemer K, Fuller-Rowell TE, Reid MC, Wells NM (2010). Environmental volunteering and health outcomes over a 20-year period. Gerontologist..

[13] Wray-Lake L, Shubert J, Lin L, Starr LR. Examining Associations Between Civic Engagement And Depressive Symptoms From Adolescence to Young Adulthood in a National U.S. Sample. Appl Dev Sci. 2017:1–13.

[14] Cicognani E, Mazzoni D, Albanesi C, Zani B (2015). Sense of community and empowerment among young people: understanding pathways from civic participation to social well-being. Voluntas.

[15] Fang S, Galambos NL, Johnson MD, Krahn HJ (2018). Happiness is the way: paths to civic engagement between young adulthood and midlife. Int J Behav Dev.

[16] Gollust SE, Rahn WM (2015). The bodies politic: chronic health conditions and voter turnout in the 2008 election. J Health Polit Policy Law.

[17] Sund R, Lahtinen H, Wass H, Mattila M, Martikainen P (2016). How voter turnout varies between different chronic conditions? A population-based register study. J Epidemiol Community Health.

[18] Nelson C, Sloan J, Chandra A (2019). Examining civic engagement Links to health.

[19] Plough A, Chandra A (2015). From vision to action: measures to movilize a culture of health.

[20] Plough A, Miller C, Tait M. Moving forward together: an update on building and measuring a culture of health. Princeton: Robert Wood Johnson Foundation; 2018.

[21] Chandra A, Miller CE, Acosta JD, Weilant S, Trujillo M, Plough A (2016). Drivers of health as a shared value: mindset, expectations, sense of community, and civic engagement. Health Aff.

[22] Karp DG (1996). Values and their effect on pro-environmental behavior. Environ Behav.

[23] Lubell M, Zahran S, Vedlitz A (2007). Collective action and citizen responses to global warming. Polit Behav.

[24] Leichter HM (2003). “Evil habits” and “personal choices”: assigning responsibility for health in the 20th century. Milbank Q.

[25] Wikler D (2002). Personal and social responsibility for health. Ethics Int Aff.

[26] Cornish F, Montenegro C, van Reisen K, Zaka F, Sevitt J (2014). Trust the process: community Health Psychology after occupy. J Health Psychol.

[27] Brown TM, Fee E (2014). Social movements in health. Annu Rev Public Health.

[28] Miranti R, Evans M (2019). Trust, sense of community, and civic engagement: lessons from Australia. J Community Psychol.

[29] Talò C, Mannarini T, Rochira A (2014). Sense of community and community participation: a meta-analytic review. Soc Indic Res.

[30] Rollero C, Tartaglia S, De PiccolI N, Ceccarini L (2009). Sociopolitical control and sense of community. A study on political participation. Psicologia Politica.

[31] Chavis DM, Wandersman A. Sense of community in the urban environment: A catalyst for participation and community development. Quarter Century Community Psychol. 2002:265–92.

[32] Carman KG, Chandra A, Weilant S, Miller C, Tait M (2019). 2018 National Survey of health attitudes: description and top-line summary data.

[33] Carman KG, Chandra A, Miller C, Trujillo MD, Yeung D, Weilant S (2016). Development of the Robert Wood Johnson Foundation National Survey of health attitudes.

[34] Chavis D, Lee K, Acosta J (2008). Sense of community index 2 (SCI-2): Background, instrument, and scoring instructions.

[35] Downs A, Blaug R, Schwarzmantel J (1957). An economic theory of democracy. Democracy: a reader.

[36] Finkel SE, Muller EN (1998). Rational choice and the dynamics of collective political action: evaluating alternative models with panel data. Am Polit Sci Rev.

[38] Quesnel-Vallee A (2007). Self-rated health: caught in the crossfire of the quest for ‘true’ health?. Int J Epidemiol.

[37] Verba S, Nie NH (1972). Participation in America: social equality and political democracy.

[39] Lewis-Beck MS, Norpoth H, Jacoby WG, Weisberg HF (2008). The American voter revisited: University of Michigan Press.

[40] Bhatti Y, Hansen KM (2012). Retiring from voting: turnout among senior voters. J Elections Public Opin Parties.

[41] Harvard School of Public Health. America’s health agenda: Priorities and performance ratings survey. Princeton: Harvard School of Public Health; 2011.

[42] U.S. Census Bureau. CPS Civic Engagement Supplement 2018 [Available from: https://www.census.gov/programs-surveys/cps/about/supplemental-surveys.html.

[43] NORC (2015). American Health Values Segmentation Study.

[44] Berezin M, Lamont M (2016). Mutuality, mobilization, and messaging for health promotion: toward collective cultural change. Soc Sci Med.

[45] Schudson M, Baykurt B (2016). How does a culture of health change? Lessons from the war on cigarettes. Soc Sci Med.

[46] Denny KJ, Doyle OM (2007). “...Take up thy bed, and vote” Measuring the relationship between voting behaviour and indicators of health. Eur J Pub Health.

[47] Blakely TA, Kennedy BP, Kawachi I (2001). Socioeconomic inequality in voting participation and self-rated health. Am J Public Health.

[48] Ojeda C (2015). Depression and political participation. Soc Sci Q.

